# Ecology and policy for exclusive breastfeeding in Colombia: a proposal

**Published:** 2012-09-30

**Authors:** Julio César Mateus Solarte

**Affiliations:** a School of Public Health, Universidad del Valle. E mail: jcmateus@fundacionfes.org

**Keywords:** Breast feeding, infant nutrition, Colombia, ecology, Health, planning

## Abstract

**Introduction:**

Breastfeeding promotion is one of the most important strategies against infant mortality and to control child undernourishment. Despite policies and plans to promote and protect breastfeeding in Colombia, its practice is low and its duration is short.

**Objective:**

To propose an ecology framework to interpret and incorporate contextual, interpersonal, and individual factors associated with the practice of breastfeeding and duration. Thereby, the plans and policies addressed to promote and protect breastfeeding in Colombia could be reinforced.

**Conclusions:**

To implement an ecology framework for Breastfeeding in Colombia, it is necessary to identify the effect of contextual factors in the biggest cultural regions of Colombia, to recognize the limitations of Infant-Friendly Hospital Initiatives to improve exclusive breastfeeding duration, to execute prospective studies in order to identify factors associated with breastfeeding duration, to design and implement plans and policies based on comprehensive planning strategies of healthcare interventions, to develop appropriate and cost-effective extra-institutional strategies aimed at prolonging the duration of breastfeeding, and to implement more reliable breastfeeding surveillance systems.

## Abstract (es)

**Introducción:**

Aumentar la práctica y la duración de la lactancia materna es una de las principales estrategias mundiales para disminuir la mortalidad infantil y controlar la desnutrición del infante menor. In Colombia, a pesar de la existencia de políticas y planes para la promoción y protección, los indicadores de lactancia permanecen en bajos niveles.

**Objetivo:**

Proponer un marco de trabajo ecológico que permita interpretar e integrar factores contextuales, interpersonales e individuales relacionados con la duración de la lactancia in Colombia. Así, se busca reforzar el diseño e implementación de políticas y planes para la promoción y protección de la lactancia materna.

**Conclusiones:**

Para la implementación de una visión ecológica de la lactancia in Colombia, es necesario esclarecer la influencia del contexto sobre la lactancia en las diferentes regiones culturales del país, reconocer las limitaciones que ha tenido la Iniciativa de Instituciones Amigas de la Mujer y la Infancia para prolongar la duración de la lactancia materna en varias comunidades, desarrollar investigaciones prospectivas para identificar factores determinantes de la práctica y la duración de la lactancia, diseñar e implementar políticas y planes basados en metodologías de planificación integral de intervenciones en salud, desarrollar intervenciones extra-institucionales apropiadas y costo-efectivas para prolongar la duración de la lactancia e implementar sistemas de vigilancia más confiables.

## Introduction

Improving nutrition of the population throughout the world, especially in developing countries, is a humanitarian and socio-economic need. To optimize the quality and quantity of foods consumed, attention must be paid to the nutritional state of all population groups, particularly pregnant women and infants, given that malnutrition may start in the uterus and extend along the vital cycle of individuals and even along the generational cycle of families, communities, or nations^1^. When deficient nutrition starts during the prenatal stage or immediate postnatal stage risk of disease or dying increases in infants and then the development and wellbeing of future adolescents and adults is impacted negatively ^2^. Maternal breastfeeding (MB) is one of the most promising alternatives to avoid deficient nutrition as of early postnatal stages. Human milk is the most appropriate food for newborns, breastfeeding benefits the health of mothers who offer it and greater practice of breastfeeding has been related with diminished infant morbidity and mortality, improvement of infant neurodevelopment, protection against chronic disease during adult life, and diminished morbidity in lactating mothers^3^. For these reasons, promotion and support of MB is one of the main worldwide strategies to decrease infant mortality and control malnutrition in young infants^4^.

The duration of MB has been the subject of multiple revisions, but increasingly scientific and political opinions admit the convenience of exclusive breastfeeding (EB) for 6 months and from then on, if it is adequately complemented, it may be extended up to two years of age and continues offering benefits to the child. In spite of this, in the national and international setting there is still a low proportion of infants fed with EB during their first six months of life^5^ ^,^ ^6^.

In Colombia, notwithstanding the existence of different government plans supporting breastfeeding for over a decade, the practice of breastfeeding is not only low but has decreased. Thus, in studies on the duration and practice of breastfeeding in mothers whose children are younger than three years of age, it was found that during the 2005-2010 period the median of duration of EB diminished from 2.2 to 1.8 months and of 46.8% who reported its practice dropping to 43.7%^7^. But when the measurement of the duration and practice of EB is done through weekly and monthly monitoring of primiparous mothers who are breastfeeding, it was found that only 1.4% practiced EB during six months and the mean duration found was 11 days (CI95%:7.1-13.0)^5^. These values place Colombia as one of the nations of median income with lower achievements in breastfeeding indicators. For this reason, it is imperative to prolong the duration and practice both of breastfeeding in general and EB in the Colombian population^7^.

Initiation and maintenance of MB is a product of the interaction of individual, family, socio-economic, political, and community factors that can be specific for a given population^8^. Thereby, it is recommended that interventions aimed at improving the indicators of breastfeeding in a population bear in mind the particularities of the community where they are implemented.

The implementation of institutional and community strategies seeking to control factors that hinder the start and continuation of breastfeeding, has managed to increase the proportion of women breastfeeding and the duration of EB has been prolonged along with MB in general. But this increase in the practice and prolongation of the duration has been different according to the community where interventions have been implemented^9^. This variability in the effectiveness of the interventions implemented constitutes evidence of the influence of the context factors upon the practice and duration of MB. Because of the aforementioned, the objective of this work was to relate, integrate, and interpret contextual, interpersonal, and individual factors related to the duration of EB in Colombia, through an ecological model that substantiates the generation or adaptation of institutional and community interventions appropriate for our setting. Additionally, this work sought to indicate a series of actions that, supported by research results, enhance the policies and plans for the promotion and protection of EB and MB in Colombia.

## An ecological-social framework for maternal breastfeeding

**Figure 1:**
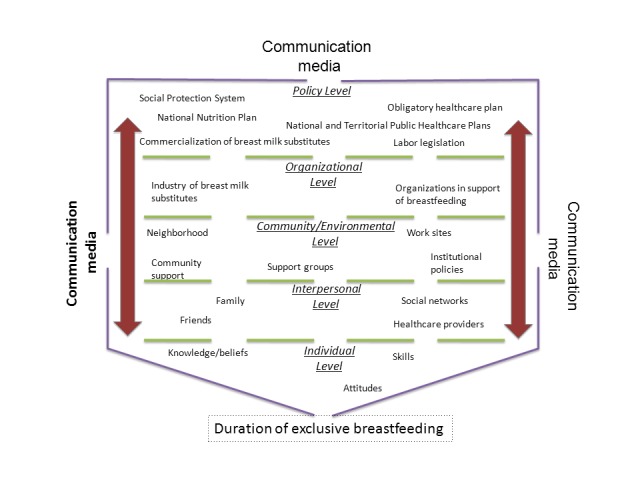
Ecology of exclusive breastfeeding in Colombia.

The number of individual, family, organizational, and community factors that can influence on the practice of breastfeeding and the complex relationships amongst them demand that the frame of the work under which is focused the promotion of maternal breastfeeding requires an integration of the different organizational levels that can be identified in a community. Based on an ecological perspective of the health promotion programs, a socio-ecological framework may be developed from which it may be illustrated how the relationship between contextual and individual factors influences women's decisions regarding MB. Figure 1 shows an application a socio-ecological vision on the Colombian case.

This socio-ecological framework presents the different levels from which women may be influenced in their decision to start and maintain breastfeeding; including from contextual to individual factors like: the role of communication media, national legislation, economic factors leading to inequality in healthcare and poverty, marketing by companies producing maternal milk substitutes, policies and practices within the healthcare institutions, community and environmental factors that support or discourage MB, family factors and of social networks, along with knowledge, beliefs and individual skills.

Mass communication media persuade and influence social norms on MB. Television programs and commercials are watched by millions daily and influence perceptions and beliefs. This can accomplish in some communities for feeding with maternal milk substitutes to be perceived as the norm and MB as the exception. Another aspect that has influenced the practice of MB is the use of the image of women's breasts with publicity and commercial purposes through which aesthetic models are established that manage to diminish the breastfeeding practice^10^.

Within the sphere of policies, the formulation, implementation, and coordination of initiatives supporting MB may produce social mobility in favor of greater practice of breastfeeding^11^. Deficient implementation, inadequate formulation, and/or low coordination of these initiatives may produce poor effects on the practice of MB or in some cases diminish its practice^8^ ^,^ ^9^.

Another factor to bear in mind within this sphere is the balance produced between the discouragement of breastfeeding that may be caused by certain commercialization policies of maternal milk substitutes and the governmental control made of said practices. In the organizational level, there is the coexistence of institutions supporting and promoting the practice of breastfeeding and the industry of milks promoting feeding with artificial formula. The two types of organizations use communication media and their political influence for their particular interests.

In the community and environmental levels there are instances where the effects of the prior levels may be reinforced or weakened. Institutions that have implemented strategies to support breastfeeding like the initiative by Women and Infant-Friendly Institutions (IAMI, for the name in Spanish) promulgated by UNICEF-WHO, have managed to modify institutional policies and practices to produce positive effects in the promotion of MB^11^. Also, institutions that have adopted marketing policies of artificial products seeking to substitute maternal milk have interfered with the breastfeeding practice among their users. Respect for labor norms in favor of breastfeeding and setting up work stations where breastfeeding may take place can also encourage or discourage the practice of breastfeeding. At the interpersonal level, the effect of significant individuals for the lactating woman like the baby's father, the grandmothers, and institutional personnel offering healthcare is well-known on the decision of starting and maintaining maternal breastfeeding. At the individual level, the woman's unawareness of one or several of the basic aspects of breastfeeding, inappropriate beliefs, negative attitudes regarding the practice, and characteristics of the newborn have been associated to late start of breastfeeding and a short duration of EB and MB in general^9^ ^,^ ^12^.

Under this framework, a woman's decision of starting and maintaining MB will depend on the mixed effect of the determinants present in each of the levels of influence in specific community. Hence, it is necessary to identify the most influential factors for the practice and duration of MB at each level to then design and implement an intervention for its promotion and protection. Additionally, identification of said factors will permit better adaptation of the strategies to a particular community. This ecological vision of breastfeeding has the logical consequence of the design of interventions that seek to influence determinant factors at the different levels of influence identified in the community. These multilevel (ecological) interventions have proven greater impact upon behaviors related to health than interventions focusing on only one level of influence^13^.

## Determinants of exclusive breastfeeding in Colombia

Consequently, by following the framework previously exposed and integrating information generated in Colombia at each of the levels, there is an ecological vision of the determinants of EB in the national setting (Fig. 1).

### Communication media:

 In Colombia, written publications targeting the general public still circulate, which erroneously present feeding with baby formula as a viable alternative to accomplish paternal participation in childcare and also present artificial milk as having equal nutritional quality as the maternal milk ^14^.

### Policy level:

 The promotion and protection of breastfeeding in Colombia has resulted in 10-year plans on breastfeeding and in policies of food safety and nutrition. Also, short-term commemorative plans like the breastfeeding week have been elaborated. But, during a regional evaluation, it was found that most of the State Social Companies^15^ had a deficient degree of implementation of the Initiative of Women and Infant-Friendly Institutions. In addition, the national guides on comprehensive attention for pregnancy and childcare mention education in breastfeeding, but do not specify a standardized methodology for said education. Also, no policies exist for continuous training or for periodic certification of the suitability of the healthcare personnel caring for pregnant women and newborns before, during, and after delivery in each of the attention levels. Apparently, the effectiveness of the national policies and plans for the protection and promotion of breastfeeding is scarce because in over a decade the breastfeeding indicators in Colombia continue in very low levels when compared with countries of similar economic development^7^.

### Organizational level:

No institution is clearly identified at the national or territorial levels, which controls the commercialization of maternal milk substitutes within healthcare institutions, although in Colombia only a few companies producing substitutes adhered voluntarily to the international code for the commercialization of said products.

### Community/environmental level:

Primiparous women from lower socio-economic levels (1 to 3) stated that once they abandoned the institution where they had given birth, they did not receive effective support from their most immediate family and social network, given that they are informed of their duty to breastfeed but are not taught how to do it or perceive sufficient support^10^. It is also possible that community violence and security factors affect the practice and duration of breastfeeding as it occurs in other communities.

### Interpersonal level:

Great discrepancy was found in a territorial study between advise on breastfeeding reported by healthcare personnel and that reported by users of childcare wards in 12 healthcare institutions^16^.

### Individual level:

National studies have been conducted showing poor knowledge and unfavorable practices toward MB among pregnant women and women in the immediate postpartum period, although most of them claim the firm intention of practicing breastfeeding^5^.

## Framework for the implementation of a socio-ecological vision of exclusive breastfeeding in Colombia

The adoption of this socio-ecological vision requires setting a framework that contemplates the appropriate identification and description of EB in our communities, identification and understanding of underlying mechanisms to the duration of EB, design and evaluation of the effectiveness and cost-effectiveness of potential interventions to prolong the duration of EB and the design, enhancement, and evaluation of national and territorial policies aimed at promoting and protecting EB.

### Appropriate identification and description of EB:

It has been described that evoking the duration of EB through interviewing mothers who have ended breastfeeding is subject to excess inaccuracies and that in one single mother inconsistencies may emerge when running the same interview on two occasions with a few weeks of difference between the first and the second run^17^. Thus, descriptions of the duration of EB based on evocation and retrospective techniques have limitations to estimate population prevalence, identify and understand the underlying mechanisms of EB, and to verify the tendency of the duration of EB in a community and the more time there is between the termination of breastfeeding and the evocation greater will the limitation be. Because of the aforementioned, it has been proposed that to avoid the inaccuracies of the evocation techniques, the measurement of the duration of EB should be based on monitoring techniques that permit studying mothers from the start to the end of EB and interviews should be made to permit inquiring on the exclusivity of breastfeeding at time intervals between one and four weeks in said women. In Colombia, prospective measurements have already begun and discrepancies were found with the retrospective measurements of the duration of EB in the city of Cali, for example^5^ ^,^ ^7^.

### Identification and understanding of underlying mechanisms to the duration of EB:

In public health, identification of the determinant factors of an event becomes the cornerstone in understanding the mechanisms that facilitate, predispose, or reinforce said event and are the essential input in the search for solutions. Given that EB is a behavior with strong individual and contextual influence, the search for and identification of its determinant factors must be supported by ecological models of health related behaviors, which recognize the existence of multiple levels from where health related behaviors may be influenced and that among factors from those levels interactions may occur. Furthermore, these models accept the specificity of each behavior and the multilevel interventions based on them are more effective.

In spite of the awareness of the usefulness of the models and theoretical frameworks to conduct research aimed at identifying determinants and understanding their mechanisms and of the support they offer to design and implement appropriate interventions^13^, their use is low and poorly availed in Latin America^18^. Colombia does not escape this situation, given that the protection and promotion plans for MB and the National Public Health Plan partly follow a model or theoretical framework for its formulation, implementation, and evaluation.

Also, most research substantiating the promotion and protection plans for breastfeeding in Colombia is transversal^7^. These types of studies do not permit adequately clarifying if a given factor was present before or after abandonment of breastfeeding or in general of the event studied. Establishing if a factor is present before ending EB is one of the indispensable conditions to involve it in the underlying mechanisms that determine its duration. This way, the factors involved in the underlying mechanisms can be of interest for an intervention aimed at promoting and protecting EB. But, when this cannot be established reliably, an intervention interested in said factor may have its effectiveness compromised.

Among the quantitative designs that permit establishing if a factor antecedes an event of interest, there are case and control studies and cohort studies. Cohort studies have an advantage over case and control studies in that measurements of EB duration are made through prospective methods and, thus, avoid bias in the information that can compromise the validity of the results. Other elements that can be of great help in identifying factors, understanding underlying mechanisms to an event and design, implement, and evaluate interventions are the qualitative methods, used as sole methodology or in combination with quantitative methods.

### Design and evaluation of the effectiveness and cost-effectiveness of potential interventions to prolong the duration of EB:

The effectiveness of an intervention of promotion may vary widely. In general, its success is quite linked to its planning before implementing it. Thus, interventions that have been subjected to a comprehensive planning process prior to their implementation are the most successful^13^. Several theoretical models exist to offer support in comprehensive planning (MCP) a promotion and prevention intervention. With these models, comprehensive planning is accomplished along with better systematization of the intervention activities^13^.

In general, all the models used in planning promotion programs follow a sequence where the initial requirement is the description and identification of the factors that can explain and predict the problems to be intervened. Then, with the information obtained from the two previous steps the contents of the intervention are defined, the implementation plan is made, and lastly, the evaluation plan is carried out. These comprehensive planning models also seek greater acceptance and sustainability of the program. For this reason, we incorporate onto these community participation criteria, institutional evaluations, strategies for community acceptance, strategies to facilitate the institutionalization of the programs, and evaluation of pertinent public policies.

Another general characteristic of these MCP is that they permit integrating theoretical frameworks that facilitate the study and understanding of the factors that predispose, reinforce, or facilitate the practice of MB, facilitate the implementation and permit better focalization of the program evaluation. Hence, with these models comprehensive interventions may be constructed coherent with the needs of the population to be intervened. Many of these MCP are supported by the principles of the practice, participation, empowerment, and development and implementation of solutions. In almost all these models participation of the community involved is fundamental, both in the definition of its problems and goals as in the development and implementation of solutions.

In the planning process, it is assumed that behaviors related to health are influenced by a vast variety of factors; thereby, the guide of a theoretical framework is necessary to define which of those factors are priorities for intervention. The systematic approach of the MCP offers specific guidelines to establish priorities; consequently, it permits for more efficient distribution of resources with more effective use.

The description and analysis of the problems contemplated in the MCP lead to awareness of the needs perceived from the community and the evaluation documents the actual needs. Identifying the needs is the starting point to construct the intervention and the evaluation of the environment, it defines the parameters in which the intervention will be implemented and will operate. Given that planning an intervention is a somewhat iterative process, the evaluation is the process by which the intervention is updated and fitted to the changing needs.

Follow up of the steps contemplated in an MCP ensures that development of interventions (programs, projects, etc.) may be replicable and that the documentation for each step of the process is available for a possible adoption or adaptation to another community. In addition, when following the sequence of the development of an MCP, the evidence that supports the progress is made explicit, crucial in arguing for an eventual healthcare policy.

## Recommendations

To implement this ecological vision of breastfeeding in Colombia, it is necessary to have a broad approach and face the methodological challenges in research, design of interventions, design and evaluation of plans and policies.

Regarding the broadening of the approach, it must be recognized that the contextual influence on the duration of EB is different in the four big Colombian cultural regions, as has occurred in other populations^12^ ^,^ ^19^ ^,^ ^20^ and that the institutional implementation of the IAMI is insufficient to significantly prolong EB duration^9^. In consequence, institutional efforts to promote breastfeeding must be complemented with the design and implementation of extra-institutional interventions of promotion and protection of breastfeeding, which are also sustainable and appropriate for our population. To accomplish broadening this approach, we must adhere to an MCP and conduct research guided by broad and integrating theoretical frameworks that permit identifying underlying mechanisms to the duration of breastfeeding in each cultural region. Given that most studies conducted in Colombia are transversal type with retrospective measurements, it is necessary for research to develop and use prospective methodologies to overcome the measurement limitations of transversal studies and to offer information with greater inferential capacity. Thus, it may be established which factors are the most determining for the practice and duration of EB. This will permit focusing the interventions on such factors and improve the efficiency of the resources invested. Regarding the evaluation of the population effects of an intervention, surveillance in public health is one of the most often used tools to indicate if population interventions are having the expected effects. In Colombia, the evaluation of progress in EB duration has been assessed via periodic transversal studies and linked methodologically^7^, but using retrospective collection techniques that for this case have questionable validity. Due to this, there is the challenge of designing and implementing a system to oversee the progress in EB duration in the nation with prospective, periodic measurements and an adequate balance among quality, utility, and costs.

Regarding the design and evaluation of plans and policies, one of the MCP available should be adopted or adapted to substantiate the content, strategies, and tools aimed at promoting and protecting breastfeeding. Additionally, the evaluation of plans and policies designed under an MCP will permit recovering and enhancing the strategies that offer benefits and reformulating those with scant effects under a logical-scientific rationality framework.

Lastly and given that a socio-ecological vision of breastfeeding permits a broad and integrating approach and demands interventions at multiple levels, a political and administrative context must be created to permit the confluence of regional and national technical and financial skills to strengthen the promotion and protection of MB in Colombia.
